# Non-additive (dominance) effects of genetic variants associated with refractive error and myopia

**DOI:** 10.1007/s00438-020-01666-w

**Published:** 2020-03-29

**Authors:** Alfred Pozarickij, Cathy Williams, Jeremy A. Guggenheim, and the UK Biobank Eye and Vision Consortium

**Affiliations:** 1 School of Optometry & Vision Sciences, Cardiff University, Maindy Road, Cardiff, CF24 4HQ UK; 2 Population Health Sciences, Bristol Medical School, University of Bristol, Bristol, UK

**Keywords:** Genetic interactions, Refractive error, UK Biobank, Complex disease

## Abstract

Genome-wide association studies (GWAS) have revealed that the genetic contribution to certain complex diseases is well-described by Fisher’s infinitesimal model in which a vast number of polymorphisms each confer a small effect. Under Fisher’s model, variants have *additive* effects both across loci and within loci. However, the latter assumption is at odds with the common observation of dominant or recessive rare alleles responsible for monogenic disorders. Here, we searched for evidence of non-additive (dominant or recessive) effects for GWAS variants known to confer susceptibility to the highly heritable quantitative trait, refractive error. Of 146 GWAS variants examined in a discovery sample of 228,423 individuals whose refractive error phenotype was inferred from their age-of-onset of spectacle wear, only 8 had even nominal evidence (*p* < 0.05) of non-additive effects. In a replication sample of 73,577 individuals who underwent direct assessment of refractive error, 1 of these 8 variants had robust independent evidence of non-additive effects (rs7829127 within *ZMAT4*, *p* = 4.76E−05) while a further 2 had suggestive evidence (rs35337422 in *RD3L*, *p* = 7.21E−03 and rs12193446 in *LAMA2*, *p* = 2.57E−02). Accounting for non-additive effects had minimal impact on the accuracy of a polygenic risk score for refractive error (*R*^2^ = 6.04% vs. 6.01%). Our findings demonstrate that very few GWAS variants for refractive error show evidence of a departure from an additive mode of action and that accounting for non-additive risk variants offers little scope to improve the accuracy of polygenic risk scores for myopia.

**Electronic supplementary material:**

The online version of this article (10.1007/s00438-020-01666-w) contains supplementary material, which is available to authorized users.

## Introduction

Myopia (nearsightedness) is an increasingly common disorder usually caused by excessive expansion and elongation of the eye during childhood (Morgan et al. 2012). The prevalence of myopia varies widely across geographical regions, peaking in urbanized areas of East and Southeast Asia and lowest in rural areas of countries with poorly developed education systems (Mountjoy et al. 2018). The expansion in size of myopic eyes is accompanied by thinning and stretching of the retina, choroid and sclera, which is associated with a heightened risk of sight-threatening impairments such as myopic maculopathy, retinal detachment and glaucoma (Ohno-Matsui 2016). The increasing prevalence of the condition coupled with its associated pathological complications has resulted in myopia becoming a leading cause of blindness and visual impairment, especially in parts of Asia (Fricke et al. 2018).

Refractive error is a continuous trait quantifying how accurately the eye focuses an image of distant objects on the retina. Myopia represents the negative arm of the refractive error distribution and hyperopia (farsightedness) the positive arm. Refractive error is highly heritable: Twin studies have reported a heritability in the range *H*^2^ = 0.75–0.91 (Sanfilippo et al. 2010), family-based studies *h*^2^ = 0.15–0.70 (Sanfilippo et al. 2010), and ‘SNP-heritability’ (inter-individual variance in refractive error explained by commonly occurring genetic variants) *h*_SNP_^2^ = 0.35–0.39% (Guggenheim et al. 2015; Shah et al. 2018). Genome-wide association studies (GWAS) have identified approximately 150 genetic variants associated with refractive error, which together explain approximately 4–6% of the inter-individual variation (Kiefer et al. 2013; Verhoeven et al. 2013; Tedja et al. 2018). Environmental and lifestyle-related risk factors such as time spent outdoors in childhood, years spent in education, birth order and time spent performing near vision tasks have been reproducibly associated with refractive error and myopia (Enthoven et al. 2019).

The known GWAS variants associated with a refractive error each have small effects when considered on a population-wide basis, i.e. each explains < 0.5% of the inter-individual variation [effects appear to be larger in certain individuals than others, most likely due to gene–environment or gene–gene interactions (Pozarickij et al. 2019)]. Current projections suggest that thousands of loci are associated with the trait (Tedja et al. 2019). In this regard, the genetic contribution to refractive error conforms closely to Fisher’s infinitesimal polygenic model (Lynch and Walsh 1997). One of the expectations or assumptions of an infinitesimal model is that the effects of individual genetic variants combine additively to determine the genetic contribution to the phenotype. Thus, for example, in predicting refractive error using a polygenic risk score (Fan et al. 2016; Enthoven et al. 2019; Ghorbani Mojarrad et al. 2020), researchers sum the effect expected for each allele of each variant according to two implicit assumptions: (1) there are no gene–gene interaction effects (epistasis), and (2) there are no allele-allele interaction effects (dominance). Dominant and recessive inheritance of monogenic traits are well known examples of non-additive allele-allele interaction effects. However, tests for dominance are rarely performed in association studies of polygenic human traits. To date, the evidence argues against widespread non-additive effects for most human complex traits (Zhu et al. 2015) although examples of common variants with strong evidence of non-additivity exist (Lenz et al. 2015; Wood et al. 2016; Plotnikov et al. 2019). If non-additive allelic effects are widespread in variants conferring susceptibility to refractive error, then accounting for them should improve the accuracy of polygenic risk scores.

Genetic variants with non-additive effects can be detected in a conventional GWAS analysis that assumes variants act additively, albeit with reduced statistical power compared to an analysis in which the correct model is specified (Sham and Purcell 2014; Dizier et al. 2017). Therefore, currently identified GWAS variants could, in fact, have dominant or recessive effects despite the assumption that they act additively. The aim of this study was to screen the known variants associated with refractive error for non-additivity (dominance).

## Materials and methods

### UK Biobank study sample

The UK Biobank is a longitudinal cohort study designed to investigate the health and well-being of older adults living in the UK (Sudlow et al. 2015). The study recruited over 500,000 adults aged 37–73 years old during the period 2006–2010. Ethical approval was obtained from the National Health Service National Research Ethics Service (Ref 11/NW/0382) and all participants provided written informed consent. Detailed information on a wide range of phenotypes was collected through questionnaire responses and physical assessments carried out at a research clinic. High-density single nucleotide polymorphism (SNP) genotyping was carried out on DNA extracted from blood samples. An ocular assessment that included non-cycloplegic autorefraction was introduced towards the end of the recruitment period, which 23% of participants underwent. Descriptions of the ophthalmic assessment and refractive error findings have been published previously (Cumberland et al. 2015; Chua et al. 2019). Details of the genotyping, imputation and quality control procedures have been reported by Bycroft et al. (2018).

### Discovery and replication sample

**Fig. 1:**
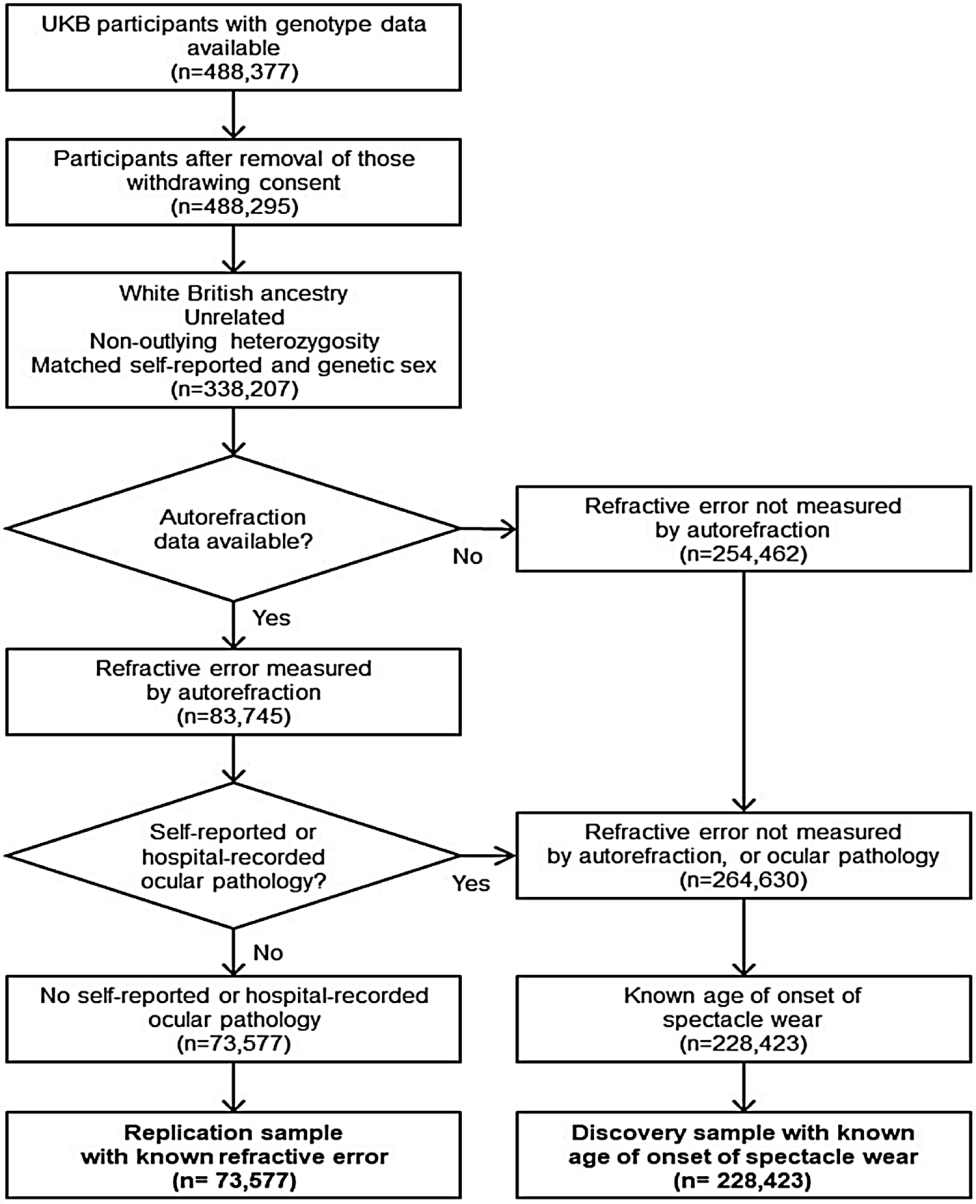
Selection of UK Biobank participants for discovery and replication sample

Separate groups of UK Biobank participants were selected as the discovery and replication samples, according to the flow diagram in Fig. 1. The replication sample comprised of *n* = 73,577 individuals classified as having White British ancestry by Bycroft et al. (2018) who underwent direct assessment of refractive error by non-cycloplegic autorefraction (Cumberland et al. 2015) and who did not self-report or have a known history of any eye disorder or pathology that may have affected their refractive error, as described (Pozarickij et al. 2019). The discovery sample comprised of *n* = 228,423 individuals of White British ancestry who had a known age-of-onset of spectacle wear (AOSW) and who either did not undergo autorefraction or who reported or had a known history of eye pathology. All *n* = 302,000 participants were unrelated, where this was defined as a pairwise kinship less than that of third-degree relatives (Bycroft et al. 2018). As with previous large-scale studies of refractive error genetics (Verhoeven et al. 2013; Tedja et al. 2018, 2019), we made the assumption that refractive error is largely stable in adulthood.

### Genetic variants associated with refractive error

We investigated 146 genetic variants with strong evidence of association with refractive error (Pozarickij et al. 2019). Specifically, these were variants identified at the threshold *p* < 5 × 10^–8^ in a GWAS mega-analysis reported by Tedja et al. (2018) that combined a meta-analysis of GWAS for refractive error in a total sample of *n* = 56,127 participants from the CREAM consortium and a meta-analysis of GWAS for age-of-onset of myopia diagnosis in a total sample of *n* = 104,293 research-consenting customers from 23andMe Inc. We restricted attention to variants that were replicated in an independent sample of *n* = 95,505 individuals from UK Biobank by Tedja et al. (2018) and that had a minor allele frequency (MAF) sufficient to expect there to be at least 50 participants homozygous for the minor allele in the replication sample (MAF > 0.03). Imputed genotype data were converted to ‘hard calls’ using the command hard-call-threshold 0.1 in PLINK 1.9 (Chang et al. 2015).

### Classification of individuals in the discovery sample as myopic or non-myopic

Supplementary Note 1 describes (i) how the relationship between refractive error and AOSW was modelled for participants in the replication cohort who had data available for both phenotypes, and (ii) how this model was utilised to infer whether participants in the discovery sample were myopic or non-myopic.

In brief, a logistic regression model was derived for participants in the replication sample with a known AOSW and a known refractive error. These participants were classified as being either myopic or non-myopic based on their known refractive error. A receiver operating characteristics curve (ROC) analysis was then undertaken to determine thresholds for classifying individuals as either myopic with 90% specificity, or as non-myopic with 90% sensitivity. Finally, the parameters from the logistic regression model were used in infer whether individuals from the discovery sample, who had a known AOSW but unknown refractive error, were myopic, non-myopic, or could not be classified.

### Statistical analysis

Analyses were carried out with R (R Development Core Team 2008). To test for genetic variants with non-additive effects, the following two logistic regression models were fitted for the participants in the discovery sample. A separate model was fitted for each of the $k$ variants:

1 $$log\left(\frac{\widehat{p}}{1-\widehat{p}}\right) \sim {\beta }^{k}{SNP}_{\text{add}}^{k}+{{\gamma }^{k}SNP}_{\text{domdev}}^{k}+{\delta }_{1}^{k}{C}_{1}+\dots +{\delta }_{m}^{k}{C}_{m}$$

2 $$log\left(\frac{\widehat{p}}{1-\widehat{p}}\right) \sim {\varphi }^{k}{SNP}_{\text{cat}}^{k}+{\delta }_{1}^{k}{C}_{1}+\dots +{\delta }_{m}^{k}{C}_{m}$$

where $\widehat{p}={\mathbb{P}}(Y=1)$ and $Y$ is the binary myopia phenotype, ${SNP}_{\text{add}}^{k}$ is the numeric count of minor alleles (0, 1 or 2) for variant *k* carried by a participant, ${SNP}_{\text{domdev}}^{k}$ is a ‘dominance deviation’ term equal to ‘1’ if a participant is heterozygous for variant *k* and equal to ‘0’ otherwise, and ${SNP}_{\text{cat}}^{k}$ is a categorical variable corresponding to the number of minor alleles for variant *k* carried by a participant coded as a factor with the heterozygous genotype class as the reference category (Table 1). A set of $m$ covariates (*C*) are included, comprising of age, age squared, gender, genotyping array (UK BiLEVE Axiom array or UK Biobank Axiom Array) and the first 10 principal components.

**Table 1:** Genotype coding for detecting non-additive effects

Term	Genotype coding (A = major allele, B = minor allele)		
	AA	AB	BB
*SNP*_add_	0	1	2
*SNP*_domdev_	0	1	0
*SNP*_cat_	Category 1	Reference category	Category 2

Variants were classified as showing nominal evidence of non-additivity in the discovery sample if *p* < 0.05 for the dominance deviation regression coefficient (${\gamma }^{k}$). Variants were classified as showing genotypic effects consistent with complete dominance or recessive action in the discovery sample if either the minor allele homozygote or major allele homozygote genotypic effects for the ${\varphi }^{k}$ regression coefficient had *p* > 0.05 in comparison with heterozygotes (in other words, *p* > 0.05 for a comparison of the trait level in the AA vs. AB genotype classes or *p* > 0.05 for a comparison of the trait level in the BB vs. AB genotype classes). Variants passing both criteria (i.e. nominal evidence of non-additivity and consistency with complete dominance or recessive effects) in the discovery sample were taken forward and tested in the replication sample using the following linear regression model (where $\widehat{y}$ is the continuous refractive error phenotype and the other terms in the equation are defined as above):

3 $$\widehat{y} \sim {\beta }^{k}{SNP}_{\text{add}}^{k}+{{\gamma }^{k}SNP}_{\text{domdev}}^{k}+{\delta }_{1}^{k}{C}_{1}+\dots +{\delta }_{m}^{k}{C}_{m}$$

A Bonferroni correction was applied to account for the number of variants tested in the replication sample.

### Polygenic risk score performance with vs. without accounting for non-additive effects

A series of three polygenic risk scores were calculated based on genotypes of the 146 variants in the 73,577 individuals with known refractive error in the replication sample. First, a ‘conventional’ polygenic risk score (*PRS* #1) was calculated using the formula:

4 $$\text{Polygenic risk score}=\sum_{k=1}^{N}{\beta }^{k}{SNP}_{\text{add}}^{k,i}$$

where ${SNP}_{\text{add}}^{k,i}$ is the numeric count of minor alleles (0, 1 or 2) carried by participant $i$ for variant $k,$ and ${\beta }^{k}$ is the log(OR) for variant *k* for association with the AOSW-inferred binary myopia phenotype in the discovery sample obtained by fitting Eq. 1 without a dominance deviation term.

A second polygenic risk score (*PRS* #2) was calculated using the formula:

5 $$\text{Polygenic risk score}= \sum_{k=1}^{N}{\beta }^{k}{SNP}_{\text{add}}^{k,i}+ \sum_{k=1}^{N}{{\gamma }^{k}SNP}_{\text{domdev}}^{k,i},$$

where $${SNP}_{\text{add}}^{k,i}$$ is the numeric count of minor alleles (0, 1 or 2) carried by participant $$i$$ for variant $$k,$$$${SNP}_{\text{domdev}}^{k,i}$$ equals ‘1’ if participant $$i$$ is heterozygous for variant *k* and equal to ‘0’ otherwise. $${\beta }^{k}$$ and $${\gamma }^{k}$$ are the additive and dominance deviation log(OR) coefficients for variant *k*, respectively, obtained by fitting the full Eq. 1 in the discovery sample.

A third polygenic risk score (*PRS* #3) was calculated exactly as for *PRS* #2 except that dominance deviation effects were only taken into account for 3 variants that were observed to show robust evidence of non-additive effects (see *Results*): *ZMAT4* variant rs7829127; *RD3L* variant rs35337422; *LAMA2* variant rs12193446. For these 3 variants, ${\beta }^{k}$ and ${\gamma }^{k}$ were taken as the additive and dominance deviation log(OR) coefficients for variant *k* in the discovery sample, fitted using Eq. 1. For the remaining 143 variants, ${\gamma }^{k}$ was taken as zero, and ${\beta }^{k}$ was the log(OR) coefficient used in *PRS* #1.

The variance in refractive error explained by each polygenic risk score was calculated by subtracting the adjusted *R*^2^ of a baseline model regressing refractive error on age and gender from the adjusted *R*^2^ of a model regressing refractive error on age, gender and polygenic risk score. The 95% confidence interval of *R*^2^ was estimated by bootstrapping (*n* = 2000 bootstraps). Polygenic risk scores were calculated using custom scripts in R.

### Simulations to assess the performance of polygenic risk scores if non-additive effects are pervasive

Genotypes for 146 variants were simulated for *n* = 75,000 individuals in a ‘training’ dataset and *n* = 75,000 individuals in a ‘test’ dataset, assuming allele frequencies matching those of the 146 genetic variants investigated above. Phenotypes for participants were calculated assuming effect sizes for the 146 variants matched those observed in UK Biobank participants (Tedja et al. 2018), under either an additive model or a dominant model, such that under both models the set of variants together explained a fixed proportion of the variance in the trait (0.02 to 0.12 in steps of 0.02). A GWAS was performed in the training dataset to estimate effect sizes assuming all variants had purely additive effects, by fitting Eq. 3*without* including a dominance deviation term. These effect sizes were used as weights for a polygenic risk score to calculate the phenotype variance explained by the 146 variants in the test dataset, by applying Eq. 4. The above GWAS and PRS analyses were then repeated accounting for both additive and dominant effects by fitting Eq. 3*with* a dominance deviation term and applying Eq. 5 to calculate the polygenic risk score. One hundred replicates were performed for each condition. R code for the simulations is given in Supplementary Note 2.

## Results

The demographic characteristics of the discovery and replication samples are summarised in Supplementary Table S1.

### Testing for variants with non-additive effects in the discovery sample

A total of 146 variants already known to be associated with refractive error from prior GWAS analyses were tested for evidence of non-additive allele-allele interaction (dominance) effects. Just 2 of the 146 variants had strong evidence of non-additive effects in the discovery sample, as judged from the *p*-value of the dominance deviation term (Eq. 2) after Bonferroni correction, *p* < 0.05/146 = 3.42 × 10^–4^. These two variants were: rs7829127 (*ZMAT4*) *p* = 8.42 × 10^–5^ and rs6420484 (*TSPAN10*) *p* = 2.07 × 10^–6^. A further 13 variants showed nominal evidence (*p* < 0.05) of dominance deviation in the discovery sample (Table 2). Of these 15 variants, 8 also had genotypic effects consistent with being fully dominant or recessive (*p* > 0.05 for a comparison of the trait level in the AA vs. AB or *p* > 0.05 for a comparison of the trait level in the BB vs. AB genotype classes) and were taken forward for testing in the replication sample. (Note that *TSPAN10* variant rs6420484 was not amongst the 8 variants taken forward since it showed evidence of incomplete dominant/recessive genotypic effects: AB vs. AA *p* = 1.95 × 10^–4^; AB vs. BB *p* = 7.56 × 10^–20^). In addition to the above analysis of variants known to be associated with refractive error, we also carried out a full GWAS analysis in the discovery sample to systematically search for variants with non-additive effects (Supplementary Note 3). However, no variant in the GWAS attained genome-wide significant evidence of a non-additive association with the trait (i.e. *p* > 5 × 10^–8^ for the dominance deviation term, for all variants; Supplementary Note 3).

**Table 2:** Tests for non-additive allelic effects in the discovery and replication samples

SNP	Gene	BAF	AOSW-inferred myopia status								Refractive error					
			Additive effect			Dominance deviation			Full dom. or rec		Additive effect			Dominance deviation		
			Beta	SE	*P* value	Beta	SE	*p* value	AB_AA *p* value	AB_BB *p* value	Beta	SE	*p* value	Beta	SE	*p* value
rs7829127	*ZMAT4*	(G) 0.20	− 0.06	0.01	4.61E−06	0.13	0.03	8.42E−05	3.99E−28	9.20E−01	0.06	0.02	9.59E−03	− 0.24	0.06	4.76E −05
rs55885222	*SNTB1*	(A) 0.39	0.06	0.01	2.45E−11	0.07	0.02	2.60E−03	6.09E−02	4.05E−08	− 0.06	0.02	2.09E−04	− 0.04	0.04	3.06E−01
rs35337422	*RD3L*	(C) 0.14	0.06	0.02	6.54E−04	0.12	0.04	5.34E−03	7.73E−01	1.26E−03	− 0.13	0.03	9.28E−05	− 0.21	0.08	7.21E−03
rs12193446	*LAMA2*	(G) 0.09	− 0.16	0.03	3.57E−08	0.16	0.06	1.03E−02	2.65E−62	1.88E−01	0.33	0.05	6.35E−11	− 0.25	0.11	2.57E−02
rs7624084	*ZBTB38*	(C) 0.45	− 0.03	0.01	2.96E−04	0.05	0.02	2.75E−02	2.34E−05	7.79E−01	0.10	0.01	4.03E−13	0.02	0.04	6.15E−01
rs17382981	*CYP26A1*	(T) 0.42	0.02	0.01	2.46E−02	− 0.05	0.02	2.86E−02	6.04E−04	6.64 E−01	− 0.06	0.01	6.01 E−05	− 0.01	0.04	7.05E−01
rs1969091	*TMC3*	(A) 0.29	− 0.02	0.01	2.36 E−02	0.05	0.03	3.52 E−02	9.93 E−06	8.09 E−01	0.10	0.02	1.11 E−07	0.08	0.05	8.78E−02
rs11101263	*FRMPD2*	(T) 0.27	0.06	0.01	3.61 E−08	− 0.05	0.03	4.69 E−02	5.94 E−14	1.48 E−01	− 0.11	0.02	1.76 E−09	− 0.10	0.05	5.23E−02

Results are presented for variants selected for testing in the replication sample (i.e. *p* < 0.05 for the dominance deviation term and *p* > 0.05 for either the AB vs. AA or AB vs. BB comparison in the AOSW-inferred myopia phenotype in the discovery sample)

*BAF* B allele frequency, *SE* standard error, *AOSW* age-of-onset of spectacle wear

### Testing for variants with non-additive effects in the replication sample

**Fig. 2:**
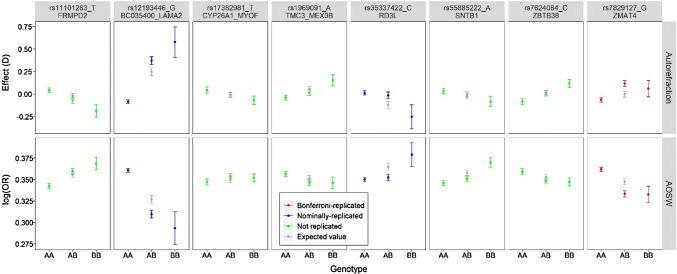
Genotypic effects in the discovery and replication samples for the eight variants with at least nominal evidence of non-additive allele-allele interaction effects in the discovery sample. Error bars represent 95% confidence interval. Grey symbols denote the effect expected in heterozygotes based on an additive model of SNP effects. The phenotype tested in the discovery sample was AOSW-inferred myopia status (lower panels), while the phenotype tested in the replication sample was autorefraction-measured refractive error (upper panels). The B allele is the minor allele

Of the 8 variants tested in the replication sample, only 1 variant (rs7829127 within *ZMAT4*, *p* = 4.76 × 10^–5^) had robust independent evidence of non-additive effects, as gauged by a dominance deviation test with Bonferroni correction for 8 tests (*p* < 0.05/8 = 6.25 × 10^–3^). This variant, rs7829127, was 1 of the 2 variants with strong evidence of non-additive effects in the discovery sample (*p* = 8.42 × 10^–5^). A further 2 of the 8 variants tested in the replication sample showed nominal evidence of non-additive effects: rs35337422 (*RD3L*) dominance deviation test *p* = 7.21 × 10^–3^ and rs12193446 (*LAMA2*) dominance deviation test *p* = 2.57 × 10^–2^. That 3 of the 8 variants exhibited at least nominal evidence of replication was more than the number expected by chance (0.4 out of 8). Results for all 8 variants are shown in Table 2 and Fig. 2.

### Polygenic risk score performance with vs. without accounting for non-additive effects

A ‘conventional’ polygenic risk score (*PRS* #1) for predicting refractive error was derived using the full set of 146 variants, under the assumption that all variants had additive effects. To avoid bias, the weights (SNP regression coefficients) for the polygenic risk score were taken by fitting Eq. 1 (but *without* a dominance deviation term) in the discovery sample. In the replication sample of 73,577 individuals with known refractive error, this conventional polygenic risk score explained 6.01% (95% CI: 5.68 to 6.34) of the variance in refractive error. A second polygenic risk score (*PRS* #2) was derived, this time accounting for both the additive and non-additive (dominance deviation) effects of all 146 variants. Weights for the polygenic risk score were taken by fitting Eq. 1 (*with* a dominance deviation term) in the discovery sample. The variance in refractive error in the replication sample explained by *PRS* #2 was 5.92% (95% CI: 5.60 to 6.24), i.e. numerically slightly lower than that obtained using the conventional polygenic risk score, but with overlapping confidence intervals. A third polygenic risk score (*PRS* #3) was derived similarly, except that account was taken of the non-additive effects of only the 3 variants showing robust evidence of non-additivity (the variants in *ZMAT4*, *RD3L* and *LAMA2*). *PRS* #3 explained 6.04% (95% CI: 5.69 to 6.36) of the variance in refractive error in the replication sample, corresponding to an extremely modest relative increase in the point estimate of variance explained compared to the conventional *PRS* #1 ((6.04–6.01)/6.01 × 100 = 0.5% improvement). Once again, the *R*^2^ estimates for *PRS* #1 and *PRS* #3 had overlapping 95% confidence intervals.

### Simulations to assess the performance of polygenic risk scores if non-additive effects are pervasive

**Fig. 3:**
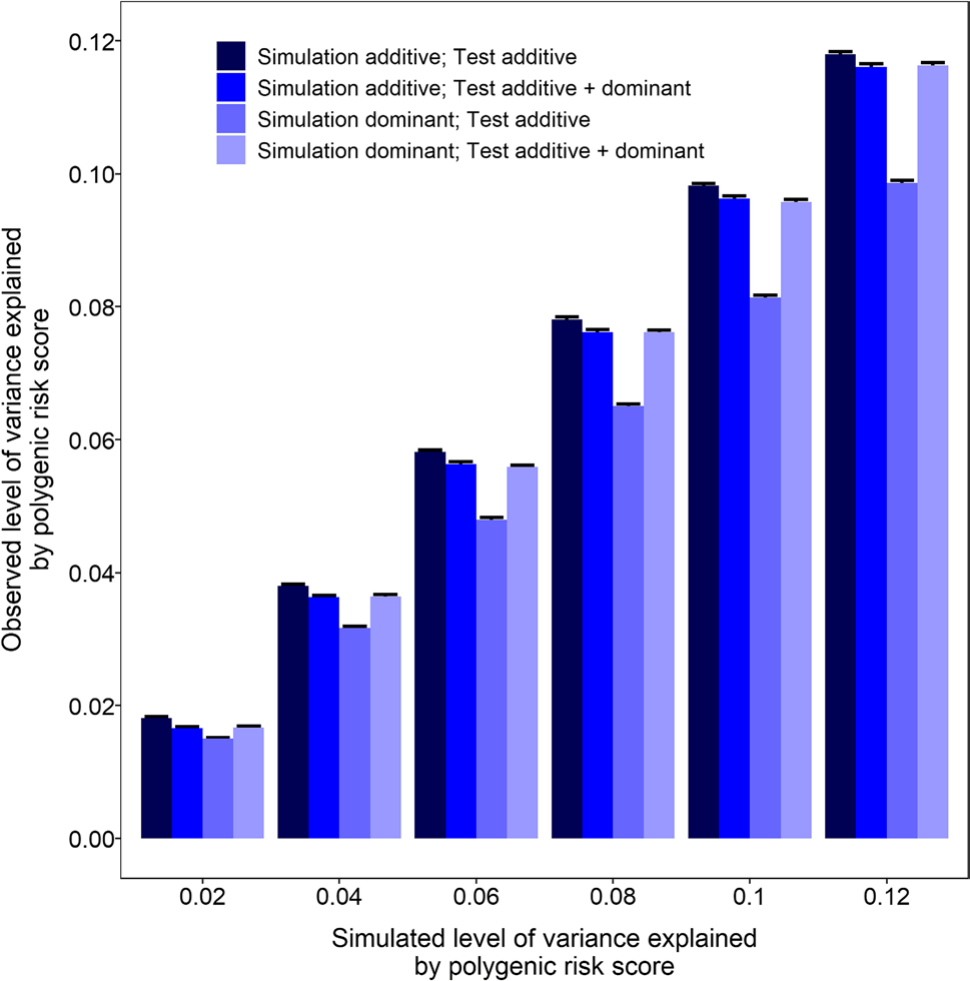
Reduction in accuracy of a 146-variant polygenic risk score if non-additive effects are pervasive. Genotypes for 146 variants were simulated in ‘training’ and ‘test’ samples of 75,000 individuals. Phenotypes were simulated assuming all variants acted additively or dominantly (labelled as ‘Simulation additive’ and ‘Simulation dominant’). Empirical variant effect sizes were estimated in the ‘training’ dataset, and then used as weights for a polygenic risk score in the ‘test’ dataset. Polygenic risk scores were calculated assuming variants acted additively (labelled ‘Test additive’) or accounting for dominance effects (labelled ‘Test additive + dominant’). The phenotypic variance explained by the 146 variants was varied from 0.02 to 0.12 in steps of 0.02. Error bars represent 95% confidence intervals from 100 replicates

Simulations were performed to gauge the reduction in accuracy of a polygenic risk score if non-additive effects were pervasive and yet were not accounted for. Specifically, genotypes and phenotypes were simulated for ‘training’ and ‘test’ samples of 146 genetic variants in 75,000 individuals under either a purely additive effects model or a purely dominant effects model. The phenotypic variance explained (*R*^2^) by the variants was set at 2% to 12% in steps of 2%. Empirical variant effect sizes were estimated by carrying out a GWAS in the ‘training’ dataset, and then these effect sizes were used as weights for a polygenic risk score in the ‘test’ dataset. The results are presented in Fig. 3. When the true model used to generate the data was purely additive, a polygenic risk score that assumed an additive model performed optimally, such that the observed *R*^2^ approached the simulated *R*^2^. In this scenario when the true model was purely additive, there was a small but consistent reduction in performance for a polygenic risk score that accounted for both additive and dominant effects. For example, when the true level of variance explained by the variants was 10%, the observed performance was *R*^2^ = 9.82% (95% CI 9.78% to 9.86%) for the additive polygenic risk score and *R*^2^ = 9.63% (95% CI 9.59% to 9.67%) for the additive + dominant polygenic risk score. In contrast, when the true model was purely dominant, then a polygenic risk score that assumed an additive model performed poorly compared to a polygenic risk score that accounted for dominance effects. For example, when the true level of variance explained by the variants was 10%, the observed performance was *R*^2^ = 8.14% (95% CI 8.10% to 8.17%) for the additive polygenic risk score and *R*^2^ = 9.57% (95% CI 9.53% to 9.61%) for the additive + dominant polygenic risk score. This corresponded to a reduction in accuracy of 14.9% caused by ignoring dominance effects (i.e. reduction in accuracy = [9.57 − 8.14]/9.57 = 14.9%). On average across all simulations, the reduction in accuracy caused by ignoring dominance effects was 14%.

## Discussion

Reproducible evidence of non-additive allele-allele interaction effects was observed for 3 of 146 variants known to be associated with refractive error (rs7829127, rs35337422 and rs12193446). By design, the pattern of allelic interaction was consistent with either fully dominant or recessive effects.

There was compelling evidence that rs7829127 had dominant/recessive effects on refractive error (since the association withstood a correction for multiple testing in both the discovery and replication samples). The variant occurs in the first intron of the *ZMAT4* gene. *ZMAT4* codes for ‘zinc finger matrin-type 4’, a gene of unknown function expressed predominantly in brain tissues. In GTEx tissue samples, rs7829127 was identified as an eQTL for *ZMAT4* expression in spleen (normalised effect size = − 0.50, *p* = 5.70 × 10^–6^, *n* = 146 samples from GTEx), yet this was not the case in GTEx brain samples. The second variant identified as likely having dominant/recessive effects, rs35337422, is a missense variant that introduces an Ile → Arg substitution in the *RD3L* gene, which codes for ‘retinal degeneration 3 like’. The variant is classed as *deleterious* by SIFT, *probably damaging* by PolyPhen, but *likely benign* by CADD. The effect of rs35337422 on *RD3L* gene expression level was not evaluated in GTEx, however variant rs8009349 is an eQTL for *RD3L* in heart tissue and is in partial LD with rs35337422 (*D*′ = 1.0, *r*^2^ = 0.10). As well as being situated within *RD3L,* rs35337422 also lies within an intron of the overlapping *TDRD9* gene (encoding ‘tudor domain containing 9’), which is implicated in male infertility. The third variant with evidence of dominant/recessive effects, rs12193446, lies within an intron towards of the 5′ end of the long *LAMA2* gene (as well as within an intron of the overlapping *LOC102723409* gene, about which little is known). The variant is not associated with effects on gene expression in GTEx samples, however, it occurs in a consensus sequence for transcription factor binding and, therefore, could potentially have a regulatory role in specific tissues. *LAMA2* encodes the alpha 2 chain of the laminin 2 protein, a component of basement membranes. Mutations in *LAMA2* are a common cause of childhood-onset muscular dystrophy, with or without occipital cortex dysgenesis (Ding et al. 2016). Of note, all 3 of the variants highlighted in the current work occur within genes rather than the inter-genic location typical of GWAS hits. Making the link between a GWAS hit and the gene through which it exerts its phenotypic effects is a major challenge in genomics. Hence, the current approach of testing for non-additive effects provides further evidence, albeit circumstantial, linking the above 3 variants to specific genes likely to have causal roles in myopia: *ZMAT4*, *RD3L* and *LAMA2*.

Differentiating a dominant from a recessive effect for a variant influencing a quantitative trait is not possible in the absence of functional molecular information. For example, purely from the genotype–phenotype information in Fig. 2, it is not clear if the minor allele (C) of rs35337422 is a dominant variant associated with an approximately + 0.25 D shift towards hyperopia or a recessive variant associated with an approximately − 0.25 D shift towards myopia. However, the functional data implicating the minor allele of rs35337422 as a deleterious missense variant for *RD3L*, makes a recessive effect shifting refractive error towards myopia the more likely option.

Variant rs6420484 was noteworthy in that there was strong evidence suggesting a non-additive association with AOSW in the discovery sample (dominance deviation *p* = 2.07 × 10^–6^), but no such evidence for non-additive effects on refractive error in the replication sample (*p* = 0.93). The minor allele of rs6420484 is a missense variant in *TSPAN10* (and is in perfect LD with deletion variant rs397693108, which is predicted to cause a frameshift in *TSPAN10*). In analyses using the same UK Biobank samples we studied here, our research group recently reported (Plotnikov et al. 2019) that the rs6420484/rs397693108 risk alleles are associated with an approximately 40–85% increased likelihood of strabismus and amblyopia, and that the variant appears to act recessively (recessive model vs. additive model, *p* = 8.10 × 10^−05^). Strabismus and amblyopia often co-occur with anisometropia and hyperopia in early childhood. Spectacles are commonly prescribed to young children to treat or manage strabismus, amblyopia and anisometropia, which would explain why we observed evidence for a non-additive association between rs6420484 and AOSW in the discovery sample here. However, as we previously reported (Plotnikov et al. 2019), the relationship between rs6420484 and *refractive error* is more consistent with an additive than a dominant or recessive mode of action, which would explain why we did *not* observe evidence for a non-additive association between refractive error and rs6420484 in the replication sample. Thus, as argued previously (Plotnikov et al. 2019), the causal variant at the *TSPAN10* locus appears to have a complex and somewhat paradoxical role in eye development, acting with a recessive mode of action as a major risk factor for strabismus and amblyopia—which often co-occurs with hyperopia—while also acting with an additive mode of action as a minor risk factor for a more myopic refractive error.

One of our primary aims when embarking on this study was to gauge the extent to which non-additive effects might impair the accuracy of polygenic risk scores for refractive error. Simulations mimicking the worst-case-scenario in which all variants actually acted dominantly, demonstrated that a ‘conventional’ polygenic risk score that assumed variants acted additively explained ~ 14% less of the phenotype variance compared to a polygenic risk score that accounted for both additive and dominance effects (Fig. 3). However, only 15 of 146 of the variants examined here displayed nominal evidence of non-additive effects in the discovery sample, and only 3 of 146 had evidence of non-additive effects in both the discovery and replication samples. Taking our findings at face value (see below), variants with non-additive effects on refractive error appeared to be scarce and our results suggested that the accuracy of a polygenic risk score for myopia is unlikely to suffer appreciably from not accounting for variants with dominant or recessive alleles (for example, the difference in performance was *R*^2^ = 6.04% vs. 6.01% when account was vs. was not taken of non-additive effects of variants in *ZMAT4*, *RD3L* and *LAMA2*). It should be stressed that the reason we detected so few variants with non-additive effects could relate to the capacity of additive models to adequately explain most of the variance of variants with dominant or recessive effects (Huang and Mackay 2016). As we were unable to robustly identify additional variants with non-additive allelic effects—despite having access to a sample of 73,577 carefully phenotyped individuals—our work suggests that the use of an additive model to derive polygenic risk scores will provide a very good approximation of the results even for variants with non-additive allelic effects. We note that the analyses we describe here only considered non-additive *allelic* effects. There is already very strong evidence that non-additive effects acting via either gene–gene or gene–environment interactions have profound effects on refractive error (Pozarickij et al. 2019); such effects would be expected to markedly impair the accuracy of polygenic risk scores for refractive error and myopia.

In summary, a set of 146 genetic variants known to be associated with refractive error were examined for non-additive allelic effects in a total sample of 302,000 participants from UK Biobank. Only one variant had strong evidence of dominant or recessive effects: rs7829127 (*p* = 4.76 × 10^–5^) situated within the first intron of *ZMAT4*. rs7829127, or a variant in high LD, is known to act as an eQTL for *ZMAT4* expression in a tissue-specific manner. Suggestive evidence also implicated another two variants at distinct loci: rs35337422, a missense variant in *RD3L* (*p* = 7.21 × 10^–3^), and rs12193446, an intronic variant in *LAMA2* (*p* = 2.57 × 10^–2^). Accounting for non-additive effects had negligible impact on the accuracy of a polygenic risk score for refractive error derived using genome-wide significant GWAS variants.

## Electronic supplementary material

Below is the link to the electronic supplementary material.

Supplementary file1 (DOCX 390 kb)

## Data Availability

Individual-level data from UK Biobank can be accessed by applying to the UK Biobank. Central Access Committee (https://www.ukbiobank.ac.uk/register-apply/).
