# Supplementary material for: Non-additive (dominance) effects of genetic variants associated with refractive error and myopia
Source: Mol Genet Genomics. 2020 Mar 29;295(4):843–53. doi: 10.1007/s00438-020-01666-w (PMC7297706; doi:10.1007/s00438-020-01666-w)
Supplement: Supplementary file 1 — Supplementary file1 (DOCX 390 kb) [file 438_2020_1666_MOESM1_ESM.docx]

**Supplementary Material**

**Non-additive effects of genetic variants associated with refractive error and myopia**

Alfred Pozarickij^1^, Cathy Williams^2^, Jeremy A. Guggenheim^1^, and the UK Biobank Eye and Vision Consortium.

^1^School of Optometry & Vision Sciences, Cardiff University, Cardiff, UK.

^2^Population Health Sciences, Bristol Medical School, University of Bristol, UK.

**Supplementary Table S1. Demographic characteristics of the discovery and replication samples.**

| **Characteristic** | **Discovery sample** | **Replication sample** |
| --- | --- | --- |
| Sample size | 228,423 | 73,577 |
| Female (%) | 124,509 (54.5%) | 38,777 (52.7%) |
| Age (95% C.I.); years | 58.3 (58.2 to 58.3) | 57.8 (57.8 to 57.9) |
| Refractive error (95% C.I.); D | Not available | -0.2 (-0.3 to -0.2) |
| Myopic^a^ (%) | 62,232 (27.2%) | 21,941 (29.8%) |
| AOSW (95% C.I.); years | 31.7 (31.6 to 31.7) | 32.4 (32.3 to 32.5) |
| ^a^ For participants in the discovery sample, myopia case/control status was inferred from AOSW, age and gender; for participants in the replication sample, myopia was defined as a refractive error ≤ ‑0.75 D.  Abbreviations: AOSW = age-of-onset of spectacle wear; D = dioptre. | | |

**Supplementary Note 1: Inferring myopia status in the discovery sample**

A 2-step process was used to infer whether individuals in the discovery sample were myopic or non-myopic. In Step 1, the relationship between refractive error and AOSW was modelled in the replication sample. In Step 2, this model was used to infer myopia status for individuals in the discovery sample.

*Step 1. Modelling the relationship between refractive error and AOSW in the replication sample*

Of the 73,577 participants in the replication sample, a total of 65,189 had information available for both refractive error and AOSW. These 65,189 participants were classified as being myopic vs. non-myopia according to the definition: refractive error <= -0.75 D. The following logistic regression model was fit using the *glm* function in R:

$${Myopic}_{i} = \mu+\left( \alpha\times{Sex}_{i} \right)+$$

$$\left( {}_{1}\times{Age}_{i} \right)+\left( {}_{2}\times{Age}_{i}^{2} \right)+ \ldots. +\left( {}_{j}\times{Age}_{i}^{6} \right)+$$

$$\left( \delta_{1}\times{AOSW}_{i} \right)+\left( \delta_{2}\times{AOSW}_{i}^{2} \right)+ \ldots. +\left( \delta_{k}\times{AOSW}_{i}^{13} \right) Eq. 1$$

Where, ${Myopic}_{i}$ is the known myopia status (1,0) of individual *i*, ${Sex}_{i}$ is a binary variable indicating the gender of individual *i*, ${Age}_{i}$ is the age in years of individual *i* when autorefraction was performed and ${AOSW}_{i}$ is the self-reported age-at-onset of spectacle wear in years for individual *i*.

The above set of 65,189 participants was randomly divided in the ratio 4:1 into a ‘training dataset’ of 51,981 individuals and a ‘testing dataset’ of 13,208 individuals. The Eq. 1 model was fit in the training dataset, and the model parameters were used to infer the probability of being myopic ($P_{myopia}$) in the testing dataset. Accuracy of inferring the correct myopia status in the testing dataset was AUROC = 0.844 (95% C.I. 0.836-0.851). The *coords* function from the *pROC* R package was then used to obtain the $P_{myopia}$threshold for obtaining at least 90% sensitivity and the $P_{myopia}$threshold for obtaining at least 90% specificity in the testing dataset. Prediction performance was poor for 25% of the testing dataset for whom both sensitivity and specificity were below 90% (Figure S1).

**Figure S1. Inferring the probability of myopia status in a ‘testing dataset’ of 13,208 UK Biobank participants with data available for AOSW and refractive error (i.e. known myopia status).** Thresholds (vertical dashed lines) are shown from ROC curve analysis to give either 90% sensitivity or 90% specificity.


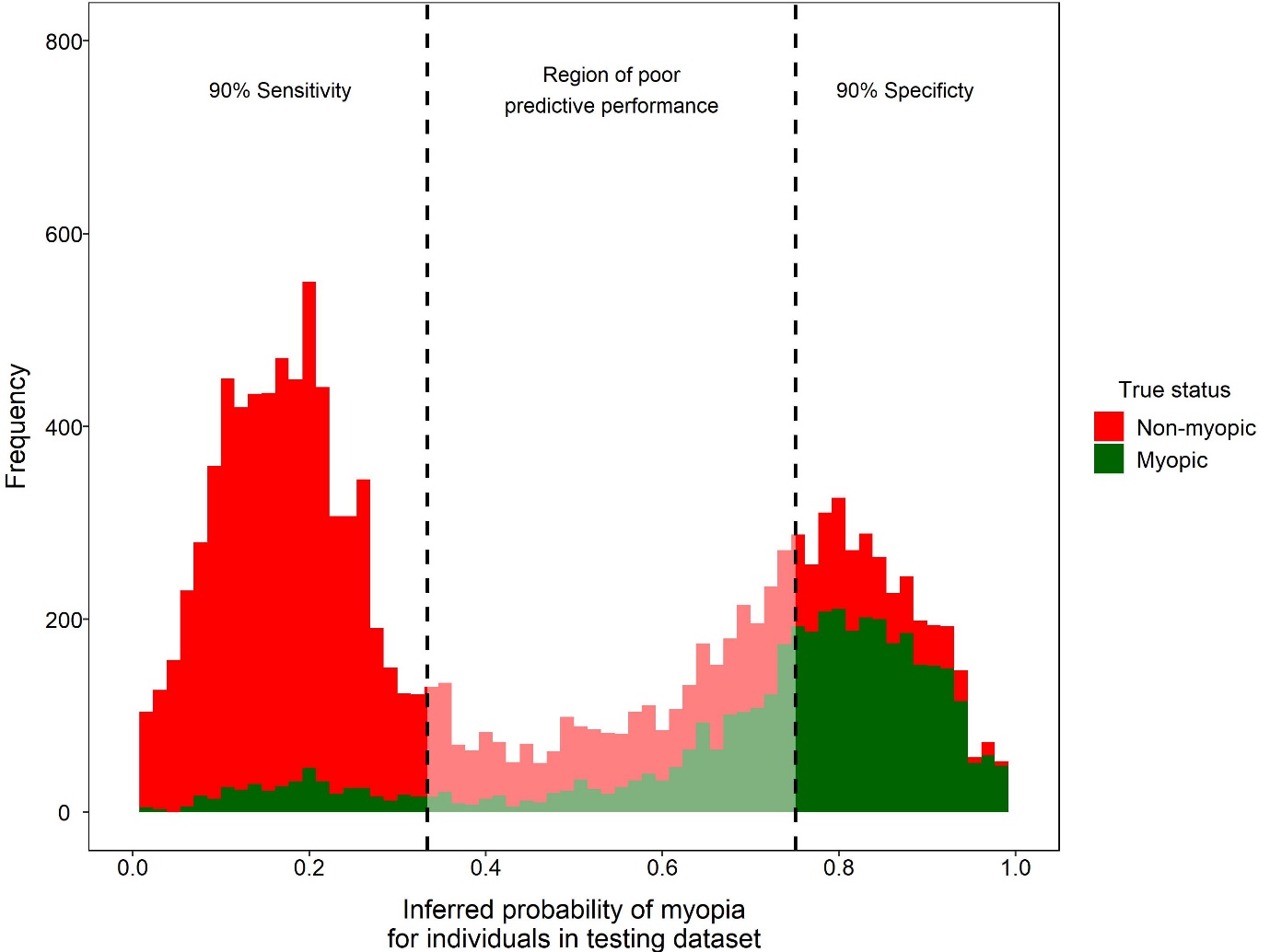


*Step 2. Inferring myopia status in the discovery sample*

The above model parameters (Eq. 1) were used to predict the probability of being myopic for participants in the discovery sample, based on their age, gender, and AOSW. Individuals in the discovery sample whose $P_{myopia}$was less than the threshold for obtaining at least 90% sensitivity were inferred to be non-myopic. Individuals in the discovery sample whose $P_{myopia}$was greater than the threshold for obtaining at least 90% specificity were inferred to be myopic. The myopia status was set as missing for those individuals whose $P_{myopia}$could not be inferred with either >90% sensitivity of >90% specificity (‘region of poor predictive performance’ in Figure S1).

**Supplementary Note 2: Simulations to assess performance of polygenic risk scores if non-additive effects are pervasive**

The R script below performs the simulations reported in the manuscript.

get_ztransform <- function(myvar){

x <- as.numeric(myvar)

m <- mean(x,na.rm=TRUE)

s <- sd(x,na.rm=TRUE)

y <- (x - m)/s

return(y)

}

get_score <- function(raw_file, add_scores, domdev_scores){

raw <- as.data.frame(raw_file)

add_weights <- as.numeric(add_scores)

domdev_weights <- as.numeric(domdev_scores)

add_weights[is.na(add_weights)] <- 0

domdev_weights[is.na(domdev_weights)] <- 0

nsnps <- length(add_weights)

scores <- as.data.frame(matrix(nrow=dim(raw)[1],ncol=nsnps))

for (snp in 1:nsnps){

add_weight <- add_weights[snp]

domdev_weight <- domdev_weights[snp]

scores[,snp] <- (raw[,snp]*add_weight) + ((raw[,snp]==1)*domdev_weight)

} # next snp

scores$score <- rowSums(scores[,1:nsnps],na.rm=TRUE)

scores$nscore <- get_ztransform(scores$score)

return(scores$nscore)

}

sim_genotypes <- function(nsnps,ninds,mafs){

nsnps <- as.numeric(nsnps)

ninds <- as.numeric(ninds)

mafs <- as.numeric(mafs)

dataGENO <- as.data.frame(matrix(ncol=nsnps, nrow=ninds))

for (snp in 1:nsnps){

maf <- mafs[snp]

dataGENO[,snp] <- rbinom(n=ninds, size=2, prob=maf)

} # next snp

return(dataGENO)

}

sim_phen <- function(dataGENO,nsnps,ninds,beta_adds,h2,model){

dataGENO <- as.data.frame(dataGENO)

nsnps <- as.numeric(nsnps)

ninds <- as.numeric(ninds)

beta_adds <- as.numeric(beta_adds)

h2 <- as.numeric(h2)

model <- as.character(model)

scores <- as.data.frame(matrix(ncol=nsnps, nrow=ninds))

if(model=="additive"){

for (snp in 1:nsnps){

scores[,snp] <- dataGENO[,snp]*beta_adds[snp]

}}

if(model=="dominant"){

for (snp in 1:nsnps){

scores[,snp] <- (dataGENO[,snp]>0)*beta_adds[snp]

}}

scores$score <- rowSums(scores[,1:nsnps],na.rm=TRUE)

scores$nscore <- get_ztransform(scores$score)

noise <- rnorm(n=ninds, mean=0, sd=1)

phens <- ((h2^0.5)*scores$nscore) + (((1-h2)^0.5)*noise)

return(phens)

}

run_gwas_get_prs_add <- function(dataTRAIN,dataTEST,nsnps,ninds,phensTRAIN){

dataTRAIN <- as.data.frame(dataTRAIN)

dataTEST <- as.data.frame(dataTEST)

nsnps <- as.numeric(nsnps)

ninds <- as.numeric(ninds)

phensTRAIN <- as.numeric(phensTRAIN)

beta_adds <- rep(NA,nsnps)

beta_domdevs <- rep(NA,nsnps)

for (snp in 1:nsnps){

mod_sum <- "fail"

mod_sum <- try(summary(lm(phensTRAIN ~ dataTRAIN[,snp])),silent=TRUE)

beta_adds[snp] <- tryCatch(mod_sum$coefficients[2,1],error = function(e) NA)

beta_domdevs[snp] <- 0

}

prs_add <- get_score(raw_file=dataTEST, add_scores=beta_adds, domdev_scores=beta_domdevs)

return(prs_add)

}

run_gwas_get_prs_dom <- function(dataTRAIN,dataTEST,nsnps,ninds,phensTRAIN){

dataTRAIN <- as.data.frame(dataTRAIN)

dataTEST <- as.data.frame(dataTEST)

nsnps <- as.numeric(nsnps)

ninds <- as.numeric(ninds)

phensTRAIN <- as.numeric(phensTRAIN)

beta_adds <- rep(NA,nsnps)

beta_domdevs <- rep(NA,nsnps)

for (snp in 1:nsnps){

snp_add <- as.numeric(dataTRAIN[,snp])

snp_domdev <- ifelse(snp_add==1,1,0)

mod_sum <- try(summary(lm(phensTRAIN ~ snp_add + snp_domdev)),silent=TRUE)

beta_adds[snp] <- tryCatch(mod_sum$coefficients[2,1],error = function(e) NA)

beta_domdevs[snp] <- tryCatch(mod_sum$coefficients[3,1],error = function(e) NA)

}

prs_domdev <- get_score(raw_file=dataTEST, add_scores=beta_adds, domdev_scores=beta_domdevs)

return(prs_domdev)

}

# Read in summary statistics for SNPs associated with refractive error

# --------------------------------------------------------------------

dataVAR <- as.data.frame(matrix(nrow=146,ncol=3))

names(dataVAR) <- c("SNP","MAF","BETA")

dataVAR$SNP <- c("rs11210537","rs11589487","rs1237670","rs11802995","rs1556867",

"rs2225986","rs1858001","rs2745953","rs11118367","rs6753137",

"rs28658452","rs17032696","rs41393947","rs10187371","rs56075542",

"rs297593","rs17428076","rs17400325","rs2573081","rs6433704",

"rs2573232","rs2573210","rs1550094","rs2276560","rs931302",

"rs9681162","rs1454776","rs4260345","rs4687586","rs7624084",

"rs13069734","rs4894529","rs7662551","rs11723482","rs7747",

"rs79953651","rs1994840","rs7667446","rs10003846","rs2166181",

"rs11952819","rs7737179","rs7449443","rs10458138","rs9295499",

"rs1207782","rs1928175","rs1150687","rs6903823","rs9395623",

"rs7744813","rs12526735","rs1064583","rs12193446","rs2326823",

"rs9388766","rs1358684","rs2116093","rs1532278","rs7829127",

"rs284818","rs3110134","rs72621438","rs72655575","rs2622646",

"rs55885222","rs10511652","rs11145465","rs7042950","rs10760673",

"rs10122788","rs11101263","rs1649068","rs9416017","rs4237285",

"rs7895108","rs745480","rs10887262","rs11202736","rs17382981",

"rs807037","rs72826094","rs511217","rs7925340","rs7941828",

"rs11602008","rs7107014","rs2155413","rs1954761","rs7122817",

"rs7933504","rs1790165","rs5442","rs7968679","rs4764038",

"rs7971334","rs117735470","rs10880855","rs3138137","rs11178469",

"rs7337610","rs1359543","rs9547035","rs9516194","rs9517964",

"rs837323","rs12883788","rs36024104","rs2855530","rs2143964",

"rs1313240","rs17125093","rs56014528","rs35337422","rs524952",

"rs34539187","rs12898755","rs6495367","rs1969091","rs79266634",

"rs28471081","rs10500355","rs56055503","rs8075280","rs2908972",

"rs115152181","rs62070229","rs4795364","rs11654644","rs12451582",

"rs8073754","rs4793501","rs7207217","rs6420484","rs10853531",

"rs12965607","rs4808962","rs235770","rs1555075","rs2229742",

"rs2823097","rs11088317","rs9680365","rs2150458","rs9606967","rs1983554")

dataVAR$MAF <- c("0.34","0.45","0.23","0.24","0.26","0.38","0.32","0.29",

"0.48","0.46","0.09","0.23","0.13","0.19","0.45","0.29",

"0.23","0.05","0.48","0.4","0.1","0.19","0.3","0.24",

"0.31","0.32","0.49","0.38","0.31","0.43","0.34","0.48",

"0.28","0.27","0.2","0.12","0.25","0.24","0.12","0.48",

"0.28","0.25","0.4","0.24","0.32","0.42","0.45","0.38",

"0.22","0.32","0.41","0.5","0.38","0.09","0.09","0.3",

"0.29","0.43","0.38","0.21","0.16","0.32","0.36","0.2",

"0.43","0.44","0.42","0.21","0.27","0.23","0.44","0.26",

"0.48","0.4","0.4","0.35","0.49","0.29","0.28","0.42",

"0.35","0.2","0.26","0.26","0.34","0.18","0.5","0.48",

"0.37","0.49","0.31","0.41","0.07","0.3","0.25","0.31",

"0.09","0.49","0.46","0.25","0.39","0.42","0.28","0.49",

"0.41","0.49","0.43","0.18","0.49","0.27","0.3","0.22",

"0.21","0.15","0.48","0.12","0.25","0.41","0.29","0.09",

"0.22","0.35","0.25","0.42","0.42","0.37","0.19","0.26",

"0.21","0.37","0.25","0.43","0.4","0.36","0.2","0.14",

"0.17","0.37","0.32","0.11","0.34","0.29","0.04","0.46",

"0.21","0.36")

dataVAR$BETA <- c("-0.10723","-0.0747093","-0.0887727","-0.0860257","-0.10881",

"-0.0934798","-0.108412","-0.0735468","-0.0871886","-0.0572376",

"-0.0847184","-0.0633908","-0.116381","-0.0397691","-0.102698",

"-0.0835047","-0.0730761","-0.256637","-0.129643","-0.12117",

"-0.15887","-0.19366","-0.195688","-0.140316","-0.0512356",

"-0.0884241","-0.0583036","-0.0545453","-0.0695639","-0.0976605",

"-0.105073","-0.0365752","-0.0955161","-0.0716368","-0.115253",

"-0.101121","-0.0837771","-0.103801","-0.11178","-0.0998742",

"-0.0323502","-0.059","-0.0369904","-0.0670767","-0.0709945",

"-0.0805458","-0.0866031","-0.0717047","0.0745578","-0.0770213",

"-0.216259","-0.0867517","-0.0880186","-0.423317","-0.230023",

"-0.047946","-0.040262","-0.0566464","-0.0483091","-0.135099",

"0.0928151","-0.0656193","-0.171569","-0.0696713","0.0834963",

"-0.0370733","-0.0964766","-0.090294","-0.115898","-0.0722829",

"-0.035898","-0.0938201","-0.0743817","-0.0569018","-0.0539976",

"-0.125619","-0.100287","-0.103053","-0.0572796","-0.0625374",

"-0.0726677","-0.109268","-0.109923","0.0723732","-0.0435415",

"-0.217495","-0.0465588","-0.0968886","-0.0971925","-0.0719488",

"-0.0503459","-0.0738437","-0.267306","-0.0761228","-0.0806785",

"-0.0294936","-0.0916732","-0.062471","-0.11557","-0.0492461",

"-0.0708414","-0.0579367","-0.0945503","-0.0436184","-0.107987",

"-0.091282","-0.0775415","-0.0981846","-0.109112","-0.105177",

"0.0977695","-0.0761325","-0.0927223","-0.0658267","-0.245382",

"-0.128213","-0.113982","-0.148815","-0.0728513","-0.113323",

"-0.111914","-0.173354","-0.0612614","-0.051859","-0.133982",

"0.0789641","-0.129414","-0.0677594","-0.0770165","-0.105755",

"0.105031","-0.0810585","-0.0433367","-0.0912285","-0.090242",

"-0.125359","-0.0820978","-0.0743802","-0.0773148","-0.118217",

"-0.0484777","-0.0602031","-0.0784776","-0.083768","-0.0393681","-0.0804198")

head(dataVAR)

# Set simulation parameters

# -------------------------

num_snps <- dim(dataVAR)[1]

num_inds <- 75000

num_reps <- 100

h2_grid <- c(0.02,0.04,0.06,0.08,0.10,0.12)

num_h2 <- length(h2_grid)

results <- as.data.frame(matrix(nrow=(num_reps*num_h2),ncol=6))

names(results) <- c("Simulated_h2","Replicate","simADD_testADD","simADD_testDOM",

"simDOM_testADD","simDOM_testDOM")

# Run simulations

# ---------------

myrow <- 0

# Loop over grid of h2 values; repeat num_rep times

for (h in 1:num_h2){

h2 <- h2_grid[h]

for (rep in 1:num_reps){

myrow <- myrow + 1

results[myrow,1] <- h2

results[myrow,2] <- rep

# Simulate genotypes for a set of SNPs with MAFs matching the imported SNPs

dataTRAIN <- sim_genotypes(nsnps=num_snps,ninds=num_inds,mafs=dataVAR$MAF)

dataTEST <- sim_genotypes(nsnps=num_snps,ninds=num_inds,mafs=dataVAR$MAF)

# Simulate phenotype under an additive model

phensTRAIN <- sim_phen(dataGENO=dataTRAIN,nsnps=num_snps,ninds=num_inds,beta_adds=dataVAR$BETA,h2=h2,model="additive")

phensTEST <- sim_phen(dataGENO=dataTEST, nsnps=num_snps,ninds=num_inds,beta_adds=dataVAR$BETA,h2=h2,model="additive")

# Standard GWAS and PRS

prs_add <- run_gwas_get_prs_add(dataTRAIN=dataTRAIN,dataTEST=dataTEST,nsnps=num_snps,ninds=num_inds,phensTRAIN=phensTRAIN)

# DOMDEV GWAS and PRS

prs_domdev <- run_gwas_get_prs_dom(dataTRAIN=dataTRAIN,dataTEST=dataTEST,nsnps=num_snps,ninds=num_inds,phensTRAIN=phensTRAIN)

results[myrow,3] <- summary(lm(phensTEST ~ prs_add))$adj.r.squared

results[myrow,4] <- summary(lm(phensTEST ~ prs_domdev))$adj.r.squared

# Simulate phenotype under a dominant model

phensTRAIN <- sim_phen(dataGENO=dataTRAIN,nsnps=num_snps,ninds=num_inds,beta_adds=dataVAR$BETA,h2=h2,model="dominant")

phensTEST <- sim_phen(dataGENO=dataTEST ,nsnps=num_snps,ninds=num_inds,beta_adds=dataVAR$BETA,h2=h2,model="dominant")

# Standard GWAS and PRS

prs_add <- run_gwas_get_prs_add(dataTRAIN=dataTRAIN,dataTEST=dataTEST,nsnps=num_snps,ninds=num_inds,phensTRAIN=phensTRAIN)

# DOMDEV GWAS and PRS

prs_domdev <- run_gwas_get_prs_dom(dataTRAIN=dataTRAIN,dataTEST=dataTEST,nsnps=num_snps,ninds=num_inds,phensTRAIN=phensTRAIN)

results[myrow,5] <- summary(lm(phensTEST ~ prs_add))$adj.r.squared

results[myrow,6] <- summary(lm(phensTEST ~ prs_domdev))$adj.r.squared

} # next rep

} # next h2

results

**Supplementary Note 3: A GWAS testing for non-additive effects in the discovery sample**

A GWAS was carried out for 5,709,726 genetic variants with MAF ≥ 0.05 and a missing call rate ≥ 0.025 in the discovery sample (Figure 1). The model in *Eq*. 2 (i.e. a model including an additive term, a dominance deviation term, and the set of covariates) was fitted using PLINK v2.00a2LM (Chang et al. 2015). For this analysis, the mean age of participants was subtracted from the age covariate and age-squared was calculated using the mean-subtracted age covariate, in order to prevent PLINK from flagging high multicollinearity between age and age-squared.

The test for additive effects provided strong evidence that numerous loci were associated with myopia (Supplementary Figures S2A and S3A), consistent with previous work (Ghorbani Mojarrad et al. 2020). However, there was no evidence suggesting an excess of variants with non-additive effects, as gauged from the distribution of *p*-values for the dominance deviation term (Supplementary Figures S2B and S3B).

These results supported the main conclusion from the analysis of 146 known GWAS variants by suggesting that variants with non-additive effects are likely to be relatively infrequent. The results also supported the approach of testing known GWAS variants rather than testing variants genome-wide, as a consequence of the high multiple-testing burden for the GWAS-based strategy.

**Supplementary Figure S2. Manhattan plots for myopia case/control GWAS analysis in the discovery sample.** Panel **A**: negative log_10_ *p*-values for the additive term (y-axis) are plotted by genomic position (x-axis). Panel **B**: negative log_10_ *p*-values for the dominance deviation term (y-axis) are plotted by genomic position (x-axis). The red line indicates *p* = 5 x 10^-8^.


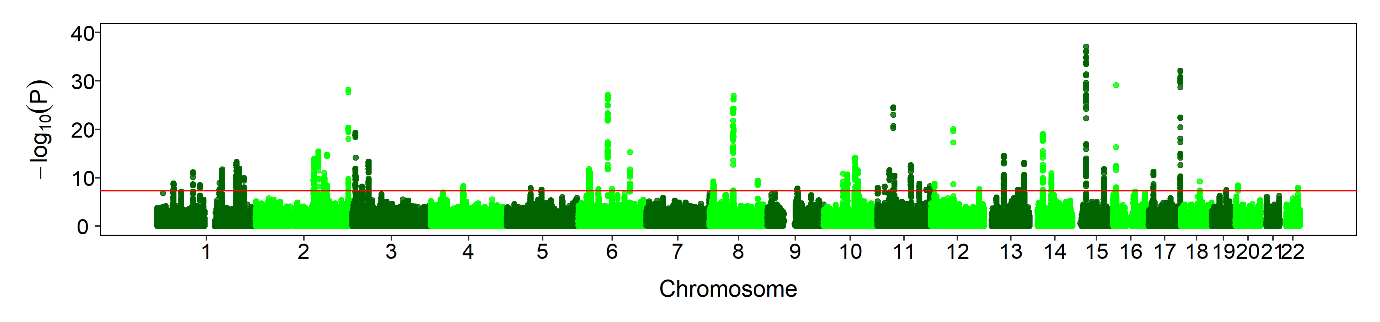
**A**

**
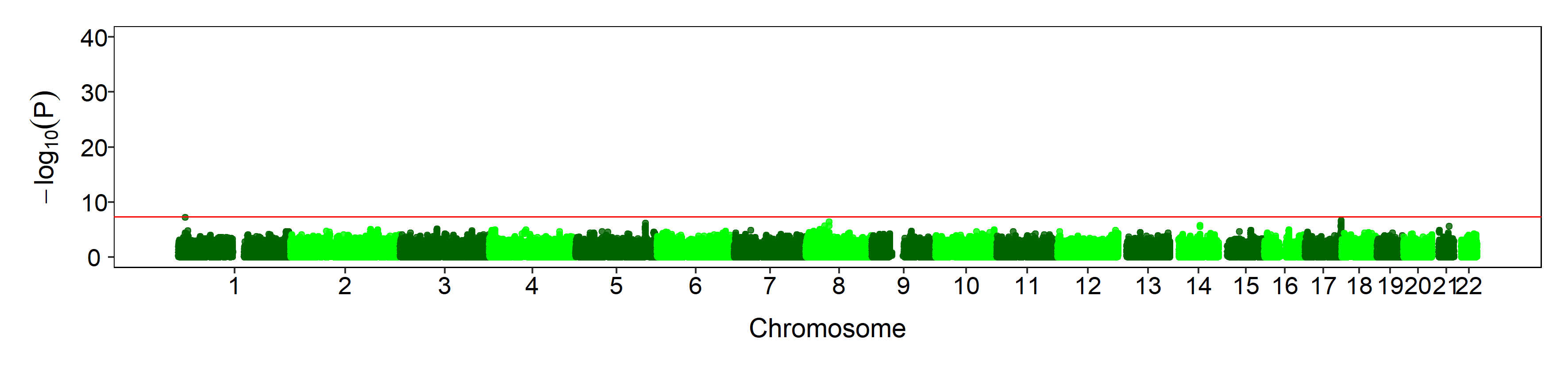
B**

**Supplementary Figure S3. Quantile-quantile plots for myopia case/control GWAS analysis in the discovery sample.** Panel **A**: Observed vs. expected negative log_10_ *p*-values for the additive term. Panel **B**: Observed vs. expected negative log_10_ *p*-values for the dominance deviation term. The red line is the line of unity (y=x) and the grey shaded region is the 95% C.I. of the distribution of *p*-values expected under the null hypothesis of no association.

**
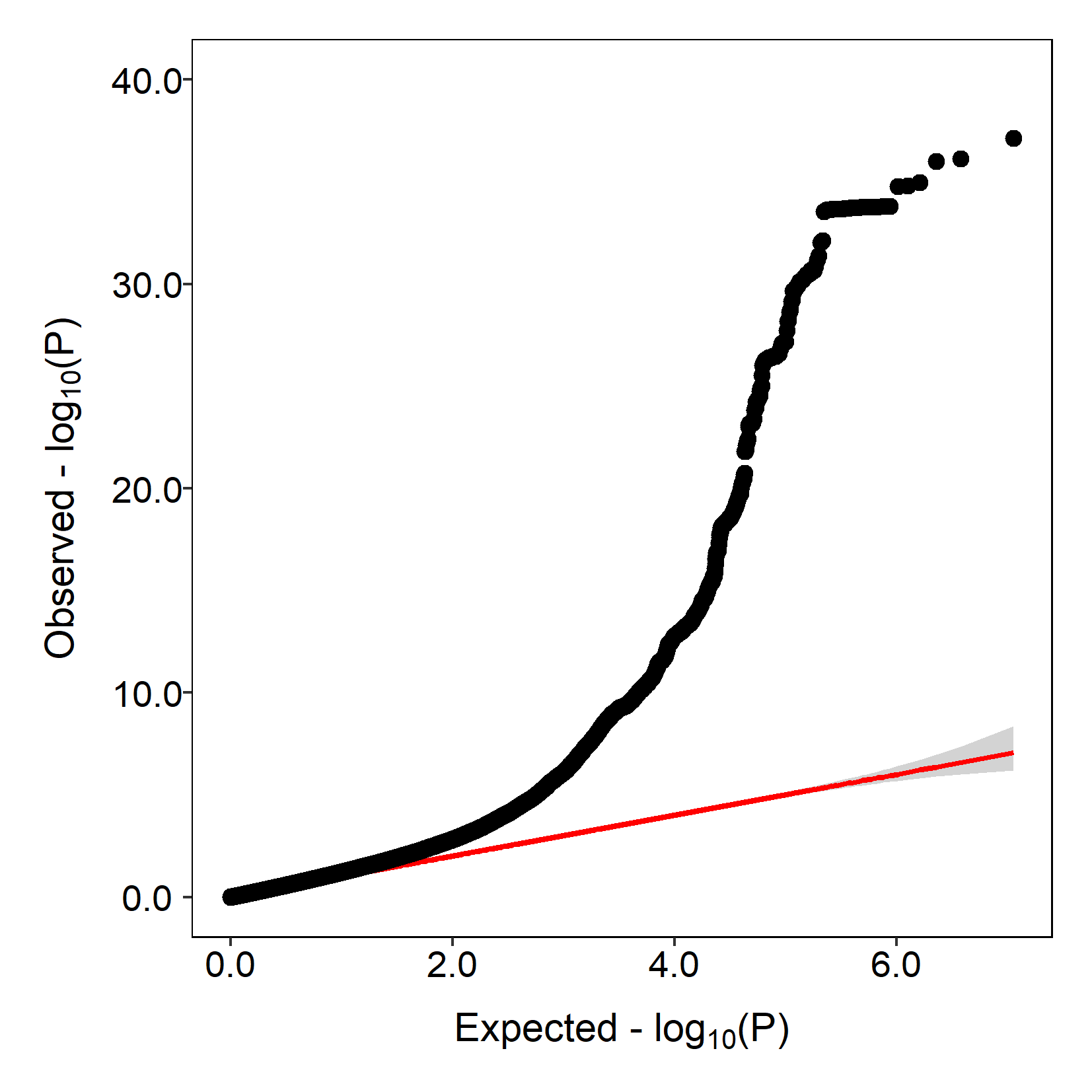
A B
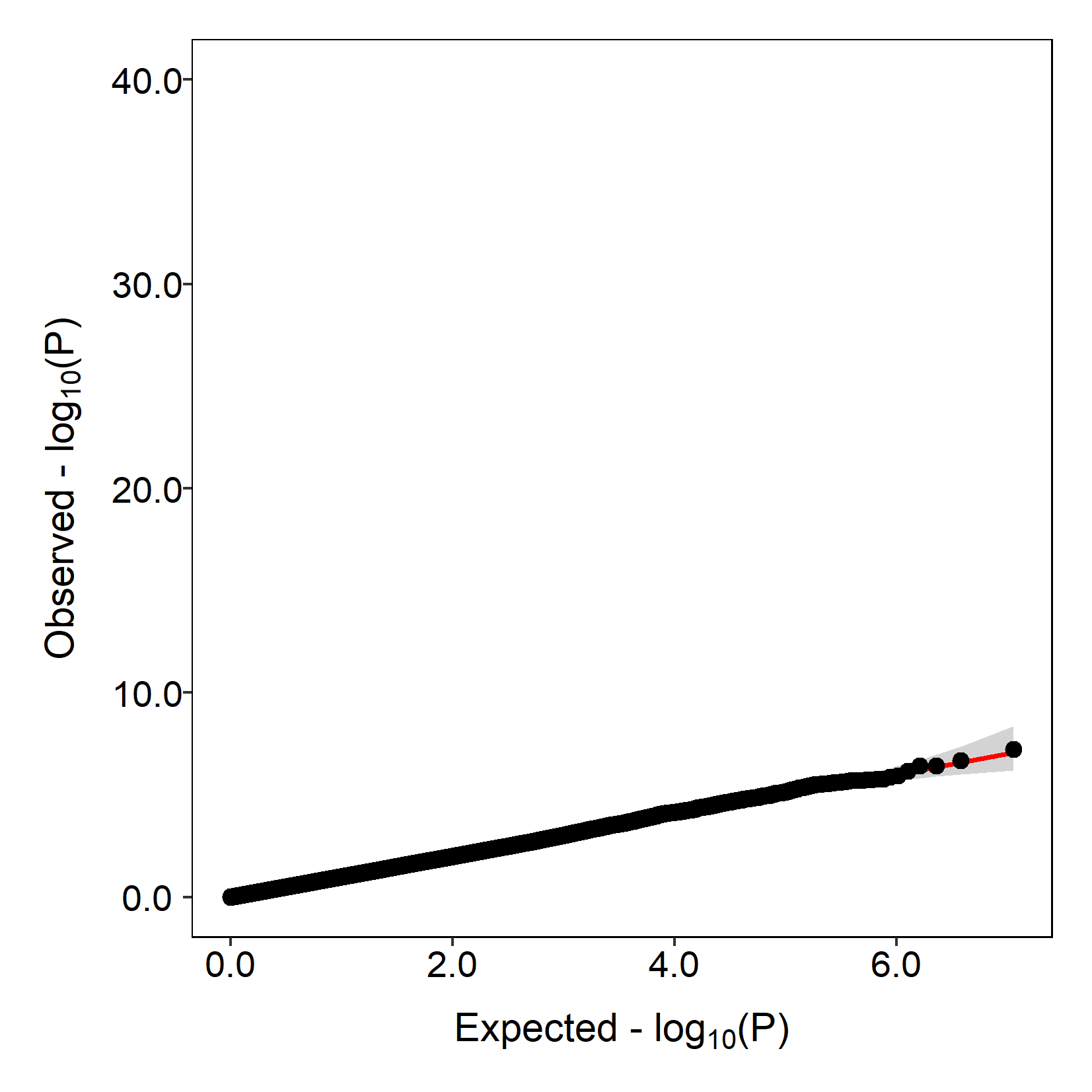
**

**References**

Chang CC, Chow CC, Tellier LC, Vattikuti S, Purcell SM, Lee JJ (2015) Second-generation PLINK: rising to the challenge of larger and richer datasets. Gigascience 4:7.

Ghorbani Mojarrad N, Plotnikov D, Williams C, Guggenheim JA, U.K. Biobank Eye & Vision Consortium (2020) Association Between Polygenic Risk Score and Risk of Myopia. JAMA Ophthalmol 138:7-13.
